# Prevalence of negative self-assessment of oral health and associated factors among adolescents from southeastern Brazil in the 2003 and 2023 Brazil Oral Health surveys

**DOI:** 10.1590/1980-549720260001.supl.1

**Published:** 2026-04-17

**Authors:** Sheyla Omonte Neves, Giana Zarbato Longo, Karla Maria Damiano Teixeira

**Affiliations:** I Universidade Federal de Viçosa – Viçosa (MG), Brazil.; II Universidade Federal do Rio Grande do Norte – Natal (RN), Brazil.

**Keywords:** Quality of life, Public health, Dentistry, Health surveys, Risk factors

## Abstract

**Objective:**

To determine the prevalence and factors associated with negative self-assessment of oral health among adolescents living in the Southeast region of Brazil.

**Methods:**

This is a cross-sectional study using data from the National Oral Health Survey of 2003 and 2023. Through examination and questionnaire, clinical, socioeconomic, and oral health perception data were obtained from 2981 individuals in 2003 and 919 in 2023. Descriptive, bivariate, and multivariate analyses were performed using Poisson regression with robust variance.

**Results:**

Negative self-assessment of oral health was observed in 43.9% (95% confidence interval — 95%CI 38.7–49.1) of participants in 2003 and 33.4% (95%CI 27.2–40.3) in 2023. In the multivariate analysis, the factors associated with worse self-assessment of oral health were: being 18 or 19 years old (prevalence ratio — PR 1.04; 95%CI 1.01–1.08 in 2003 and PR 1.27; 95%CI 1.00–1.63 in 2023), not owning a home (PR 1.16; 95%CI 1.01–1.32 in 2003), receiving government assistance (PR 1.40; 95%CI 1.11–1.77), feeling the need for treatment (PR 2.7; 95%CI 1.94–3.74 in 2003 and PR 3.0; 95%CI 1.58–5.70 in 2023), experiencing toothache (PR 1.67; 95%CI 1.38–2.04 in 2003 and PR 1.72; 95%CI 1.17–2.54 in 2023), having dental calculus (PR 1.22; 95%CI 1.04–1.43 in 2003 and PR 1.84; 95%CI 1.28–2.66 in 2023), having missing, decayed or filled teeth (PR 1.02; 95%CI 1.01–1.04 in 2003), loosing teeth due to cavities (PR 2.19; 95%CI 1.42–3.37 in 2023), and last visiting the dentist two to three years earlier (PR 1.89; 95%CI 1.39–2.56 in 2023).

**Conclusion:**

A high prevalence of negative self-assessment of oral health was observed among adolescents in the Southeast region, a finding associated with a multidimensional structure of variables.

## INTRODUCTION

Self-rated health is a subjective variable related to clinical conditions and indicators of morbidity and mortality^1^. It is a simple way to understand the multidimensionality related to an individual's perspective on their own health^2^. In the context of oral health, self-assessment reflects Oral Health-Related Quality of Life (OHRQoL), associating it with the objective condition of health and functionality, in addition to incorporating cultural, political, economic, and social values^1,3–8^. Thus, oral health is viewed more broadly, recognizing its complexity and the impact of oral health inequalities and associated social determinants^6^. The literature shows that non-whites^9^, with a per capita household income of up to one minimum wage^10^, and less privileged social classes^10,11^ have a higher proportion of negative health assessments in the Brazilian population. Studies also highlight that socioeconomic conditions are important determinants of population health^10,11^.

In 2004, the implementation of the Brasil Sorridente (Smiling Brazil) program expanded Brazilians’ access to oral health services^12^. Analysis of the cycle of this policy points to advances in health indicators, expansion of the dental service network, and increased healthcare coverage^12^. On the other hand, the compromise in quality of life, reflected in self-assessment of oral health, remained high in studies conducted after the implementation of Brasil Sorridente^4,5,13,14^.

In this context, self-assessment of oral health can be a significant complement to clinical indices, as it considers the biological, socioeconomic, and cultural elements that impact oral health care and the health-disease process^1,10,14^ . This variable is particularly relevant in adolescence, a stage of life when responsibility for self-care generally begins, and eating and hygiene habits are consolidated^15^. In addition, as it is a transitional phase marked by the intertwining of dependence and independence, adolescence favors the emergence of behaviors that are potentially harmful to health^6^. Furthermore, conditions that negatively affect young people's well-being can have repercussions on their families’ quality of life^16^.

It should be noted that regional heterogeneity, with its cultural and socioeconomic differences, compromises the generalization of national results for individuals in a particular region of the country^10^ . This reinforces the importance of regionalized health information. Thus, the objective of this study was to assess the prevalence of negative self-assessment of oral health (NSASOH) among Brazilian youth aged 15 to 19 years living in the Southeast region, and to investigate its association with clinical, demographic, predisposing/facilitating conditions (such as income and health care), perception, and need for treatment. The null hypothesis is that these factors do not influence young people's self-assessment of oral health. There are studies conducted in other regions of the country and in other age groups on the subject^4,5,13,14^, but this is the first to analyze young people in the Southeast. Furthermore, the literature points out that this topic is still under-explored in national and international research^4^, which reinforces the importance of this investigation.

## METHODS

This is a cross-sectional study that included individuals aged 15 to 19 years, residing in southeastern Brazil, who participated in the 2003 (SBBrasil 2003) or 2023 (SBBrasil 2023) Brazilian Oral Health Survey (SBBrasil). Those whose data on self-assessment of oral health were incomplete were excluded. The SBBrasil Project has national coverage and is representative of state capitals, the Federal District, and the five geographic regions (North, Northeast, Southeast, South, and Midwest). The planning considered the selection of a probabilistic sample by clusters. Training and calibration of dental teams were carried out. Details on design, sample calculation, calibration, and data collection can be found in the official reports^17,18^. Data collection took place between 2002 and 2003^17^ and from 2022 to 2024^18^. SBBrasil 2003 was approved by the National Research Ethics Commission (Conep), according to opinion n° 581/2000^17^. SBBrasil 2023 was approved according to Conep opinion n° 4.823.054/2021^18^.

The oral conditions examined included: caries prevalence using the Decayed, Missing, and Filled Teeth (DMFT) index; periodontal condition using the Community Periodontal Index (CPI), measuring bleeding, calculus, and the presence of periodontal pockets; and dental occlusion via the Dental Aesthetic Index (DAI), which combines occlusal status and orthodontic need^17,18^. Socioeconomic, demographic, self-reported oral morbidity, access/use of oral health services, and self-perceived oral health data were also collected through a questionnaire administered during the home visit^17,18^.

The dependent variable was self-assessment of oral health. In 2003, it was assessed by the question: "*How would you rate your oral health?*", with five response options: "very poor," "poor," "fair," "good," and "excellent." Responses such as "no information" or "don't know/did not report" were excluded from the analysis. In 2023, the question asked was: *"In general, how do you rate your oral health (teeth and gums)?*", with the following responses: "very good," "good," "fair," "poor," "very poor," and "don't know/did not respond," the latter being excluded from the analysis. For analytical purposes, the responses were grouped into two categories: positive (excellent, very good, or good) and negative (fair, poor, very poor), as used in previous studies^1,4,8,13^. There is a marked difference in the question and answer options regarding self-assessment of oral health in the 2010 SBBrasil questionnaire compared to other SBBrasil surveys. Thus, we chose not to analyze the 2010 SBBrasil data in this study.

The independent variables in this study were selected based on the hierarchical model of Gift et al.^19^, with minor adaptations, as other authors have done^5^. The conceptual model theorizes that self-assessment of oral health is a multiple function, including demographic characteristics, predisposing/facilitating factors, oral health condition factors, and self-perception factors of the need for treatment^19^.

**Figure 1:**
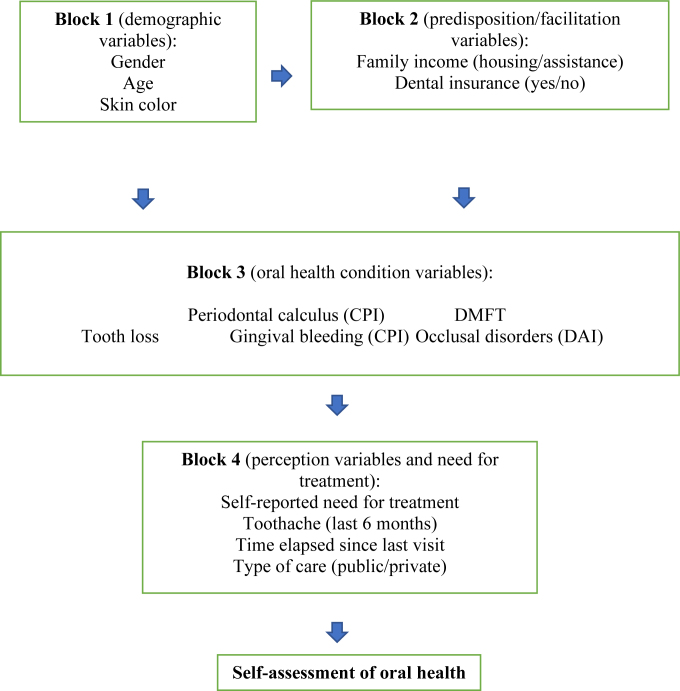
Theoretical model for determining independent variables adapted from the model proposed by Gift et al.^19^

Similarly, the independent variables in this study were organized into four blocks (Figure 1):

Blocks 1 and 2: demographic and predisposing/facilitating variables;

Block 3: clinical oral health conditions; and

Block 4: variables related to treatment need and perception.

Of the more than 30 indicators available in the SBBrasil 2003 and SBBrasil 2023 databases, the variables most representative of oral health and health-related quality of life (HRQoL) were selected, considering the current literature and the conceptual model adopted, which includes the social and biological determinants of health^5,19^.

Statistical analysis was performed using Stata^®^ software, version 17.0, with the *survey* module, following the recommended guidelines for complex samples^20^. Initially, the sample was described, presenting proportions, means, and 95% confidence intervals (95% CI). Next, bivariate analyses of the prevalence of the outcome were conducted, considering negative self-assessment as the reference category of the dependent variable, calculating the prevalence ratios (PR) and 95% CI. To test the effect of the independent variables on the outcome, the Poisson regression model with robust variance was used, which is indicated when the prevalence of the outcome exceeds 20%^21^. In the simple analysis, the association between each independent variable and the outcome was verified, calculating PR and 95% CI. Variables with a p-value <0.25 in the crude analyses were included in the multiple model^22^. At the end of the multiple analysis, variables with p<0.05 remained in the final model for each block and were considered adjustment factors for subsequent blocks, without being accompanied by a deviance or Pearson test to assess adjustment.

### Data availability statement

Data available upon request: The entire dataset supporting the results of this study is available upon request from the Ministry of Health. To access the SB Brasil databases, it is necessary to agree to the terms of commitment and fill in the data requested by the Ministry of Health.

## RESULTS

The SBBrasil Project collected data from 16,832 and 8,054 individuals aged 15 to 19 years in SBBrasil 2003 and 2023, respectively. In the Southeast region, the sample consisted of 2,981 adolescents in 2003 and 919 in 2023. Of these, 86 individuals were excluded in 2003 (incomplete information regarding self-assessment of oral health), resulting in a sample of 2,895 adolescents (97.1%). In 2023, 21 individuals were excluded for the same reason, totaling 898 individuals included in the analysis (97.7%).

Table 1 shows the prevalence of negative oral health self-assessment (NSOHS), which was 43.9% (95%CI 38.7–49.1) in the participants of SBBrasil 2003 and 33.4% (95%CI 27.2–40.3) in SBBrasil 2023. In the most recent year, 35.1% (95%CI 27.9–43) of adolescents belonged to families benefiting from income transfer programs, such as Bolsa Família or the Continuous Cash Benefit (BPC), a proportion similar to that observed among those who did not own their own home in 2003 (20.3%; 95%CI 14.5–28.1).

**Table 1:** Distribution and prevalence of negative oral health self-assessment (NSOHS) according to characteristics of the sample of adolescents (15 to 19 years old) in the periods 2003 and 2023. SBBrasil Project, MS, Brazil.

		Sample distribution (n=2,895)		NSOHS prevalence		Sample distribution (n=898)		Prevalence of NSOHS	
		2003				2023			
		Average %	95%CI	%-average	95%CI	%-mean	95%CI	%-mean	95%CI
Total				43.9	38.7–49.1			33.4	27.2–40.3
Gender									
	Male	42.2	37.6–46.9	43.1	37.1–49.3	51.3	45.3–57.2	33.2	22.2–46.3
	Female	57.8	53.1–62.3	44.3	39.2–49.6	48.6	42.7–54.6	33.7	26.8–41.3
Age (years)									
	15–17	64.3	59.9–68.4	39.2	33.8–44.8	65.7	57.3–73.1	29.8	20.7–41
	18–19	35.7	31.6–40	52.2	46.3–58.1	34.3	26.9–42.6	40.3	33.4–47.8
Skin color									
	White	41.1	31.9–50.8	40.2	33.4–47.2	46	36–56.1	27.5	19.4–37.5
	Non-white	58.9	49.2–68.1	46.4	40.9–52	54	43.8–63.9	38.2	31.7–45.1
Receives benefits									
	No	-	-	-	-	64.8	56.9–72	26.6	20.8–33.2
	Yes					35.1	27.9–43	46.9	37.4–56.7
Own home									
	Yes	79.7	72.4–85.5	43.4	38.5–48.3	-	-	-	-
	No	20.3	14.5–28.1	45.8	36.6–55.2				
Dental plan									
	Yes	9.7	7.5–12.4	42.9	30.1–56.7	16.9	9.9–27.3	18.8	11.8–28.8
	No	90.3	87.6–92.4	43.9	38.8–49.3	83.1	72.7–90.1	36.7	29.2–44.9
Missing teeth									
	None	80.2	77–83	39.6	33.2–46.4	92.3	88.8–94.7	30.3	24.4–36.9
	1 or more	19.8	16.9–23	61	55.2–66.5	7.7	5.2–11.2	71.2	54.5–83.6
Dental calculus									
	Healthy sextants	65.8	54.2–75.8	36.5	29.9–43.6	67.8	59.5–75.1	24.6	19.7–30.3
	Presence of calculus	34.2	24.2–45.8	54.1	47.9–60.3	32.1	24.8–40.5	51.5	38.9–63.9
Gingival bleeding									
	Healthy sextants	74.6	63.6–84.2	36.5	30–43.6	68.9	61.4–75.6	27.3	20.6–35.1
	Presence of bleeding	25.4	16.7–36.4	49.3	41.5–57.1	31	24.4–38.6	46.9	38.4–55.7
	DMFT (mean)	4.9	4.5–5.4	5.8	5.3–6.3	2.8	2.2–3.5	4.1	2.8–5.5
DAI									
	Without occlusal disease	43.5	38.4–48.8	44.9	38–51.9	72.5	65.2–78.1	30.1	23.2–38.1
	Defined occlusopathy	22.5	20.1–25	39.9	34.8–45.1	14	9.8–19.7	36.3	21.8–53.7
	Severe occlusal disorder	14.9	12.3–17.8	44.8	38.7–51.1	6.1	4.4–8.3	34.2	22.1–48.8
	Very severe	19.1	16.9–21.5	45.2	35.3–55.6	7.3	4.5–11.6	58.5	41.6–73.6
Need for treatment									
	No	26.3	20.7–32.7	17.8	12.9–23.8	40.8	32.3–49.8	11.7	6.6–19.9
	Yes	73.7	67.2–79.3	53.2	47.7–58.5	59.2	50.1–67.7	48.9	41.5–56.3
Time (years)*									
	Up to 1	44.6	39.5–49.8	35.3	29–42.1	53.4	45.6–60.9	25.9	18.3–35.2
	2 to 3	24	19.3–29.4	47.5	41.8–53.3	35.1	27.4–43.7	42.5	34.4–51
	More than 3 or never	31.4	27.2–36.2	53.3	46.0–60.4	11.5	7.8–16.7	34.6	23.5–47.7
Type of service†									
	Private	38.4	34.8–42.1	41.6	34.7–48.8	61.9	56.2–67.4	27.9	20.2–37.2
	Public	61.6	57.9–65.2	44.2	39–48.5	38	32.6–43.8	40.5	29.5–53
Pain (last 6 months)									
	No	64.3	58.6–69.5	32.3	26.5–38.7	80.9	75.8–85.1	27.6	22.1–33.7
	Yes	35.7	30.5–41.4	64.7	59.2–69.8	19.1	14.8–24.2	58.7	37.3–77.2

NSOHS: Negative Self-Assessment of Oral Health; DMFT: Decayed, Missing, and Filled Teeth Index; DAI: Dental Aesthetic Index.

* time elapsed since last dental appointment;

† type of service used at last dental appointment.

Table 2 shows the crude and adjusted prevalence ratios for each independent variable, as well as the models obtained in the multiple analysis, stratified by study year. In 2003, after adjusting for the variables in Block 1, adolescents aged 18–19 years had a higher prevalence of negative self-assessment of oral health. In Block 2, after mutual adjustment and control for the variables in Block 1, the absence of own housing remained associated with the outcome. Among the clinical variables (Block 3), having missing, decayed, and filled teeth and the presence of dental calculus remained significantly associated with negative perception of oral health. In Block 4, reporting toothache in the last six months and self-reported need for treatment were statistically significantly associated even after adjustments.

**Table 2:** Univariate and adjusted analyses, prevalence ratios, and 95% confidence intervals obtained by Poisson regression analysis for the association between negative self-assessment of oral health and independent variables in the sample of adolescents (aged 15 to 19 years) in the periods 2003 and 2023. SBBrasil Project, MS, Brazil

Variables		2003		2023	
		Crude OR (95%CI)	Adjusted PR (95%CI)	Crude PR (95%CI)	Adjusted PR (95%CI)
Gender					
	Male	1		1	
	Female	1.02 (0.94–1.13)		1.01 (0.64–1.61)	
Age (years)					
	15–17	1	1	1	1
	18–19	1.10 (1.07–1.1.4)	1.04 (1.01–1.08)	1.35 (0.88–2.07)	1.27 (1.00–1.62)
Skin color					
	White	1		1	
	Non-white	1.16 (0.98–1.36)		1.38 (0.97–1.97)	
Receives benefits					
	No			1	1
	Yes			1.77 (1.37–2.27)	1.40 (1.11–1.77)
Own home					
	Yes	1	1		
	No	1.06 (0.93–1.25)	1.16 (1.01–1.32)		
Dental plan					
	Yes	1		1	
	No	1.03 (0.75–1.40)		1.95 (1.08–3.49)	
Missing teeth					
	None	1		1	1
	1 or more	1.54 (1.26–1.88)		2.35 (1.77–3.11)	2.19 (1.42–3.37)
Dental calculus					
	Healthy sextants	1	1	1	1
	Presence of calculus	1.48 (1.22–1.80)	1.22 (1.04–1.43)	2.09 (1.51–2.90)	1.84 (1.28–2.66)
Gingival bleeding					
	Healthy sextants	1		1	
	Presence of bleeding	1.35 (1.08–1.68)		1.72 (1.27–2.32)	
DMFT (mean)		1 1.04 (1.02–1.06)	1 1.02 (1.01–1.04)	1 1.06 (1.03–1.09)	
DAI					
	No occlusopathy	1		1	
	Defined occlusopathy	0.89 (0.76–1.04)		1.20 (0.74–1.95)	
	Severe occlusal disorder	0.99 (0.83–1.20)		1.13 (0.72–1.81)	
	Very severe	1.01 (0.78–1.29)		1.94 (1.33–2.84)	
Need for treatment					
	No	1	1	1	1
	Yes	2.99 (2.22–4.03)	2.70 (1.94–3.74)	4.17 (2.31–7.53)	3.00 (1.58–5.70)
Time (years)*					
	Up to 1	1		1	1
	2 to 3	1.34 (1.12–1.61)		1.64 (1.21–2.23)	1.89 (1.39–2.56)
	> 3 or never	1.51 (1.26–1.80)		1.34 (0.86–2.08)	1.16 (0.76–1.77)
Type of service†					
	Private	1		1	
	Public	1.06 (0.92–1.23)		1.45 (0.97–2.15)	
Toothache					
	No	1	1	1	1
	Yes	2.00 (1.63–2.47)	1.67 (1.38–2.04)	2.13 (1.48–3.05)	1.72 (1.17–2.54)

PR: Prevalence Ratio; 95% CI: 95% Confidence Interval; DMFT: Decayed, Missing, and Filled Teeth index; DAI: Dental Aesthetic Index.

* time elapsed since last dental visit;

† type of service used during last dental visit.

In 2023, the variables age (18 or 19 years), receipt of government assistance, tooth loss due to caries, presence of dental calculus, perceived need for dental treatment, time since last dental visit (two to three years), and recent toothache were positively associated with dissatisfaction with oral health, with statistical significance, as shown in Table 2.

## DISCUSSION

With regard to young people aged 15 to 19 living in the Southeast region of Brazil, the data presented show a decline in important indicators from 2003 to 2023, such as the Decayed, Missing, and Filled Teeth (DMFT) index and dental pain, in line with the successful implementation of Brasil Sorridente, which has brought about structural and organizational improvements in the oral health system in its 20 years of existence^12^. However, the prevalence of NSOHS remained above 30%. Nevertheless, there was a decline in this variable (from 43.9% in 2003 to 33.4% in 2023), but without statistical significance. Studies conducted on adolescents in other regions, such as in the cities of Santa Maria (RS)^4^and Manaus (AM)^14^, also observed high dissatisfaction with oral health, suggesting that, despite structural and care advances, subjective indicators among adolescents remain critical.

However, studies with adults and the elderly generally present more positive self-assessments^1,5,13,23^, which may reflect methodological differences or, more likely, different perceptual constructions of health throughout the life cycle. Emotional, cultural, and social factors modulate these perceptions, which are more acutely felt during adolescence, a period of intense psychosocial changes^15^. Thus, it is argued that the advances achieved by Smiling Brazil need to be accompanied by strategies that incorporate the subjective determinants of oral health, especially in youth.

Among the demographic variables (Block 1), age remained significantly associated with the outcome in the adjusted model. The prevalence of NSOHS increased among older adolescents (18–19 years), replicating previous findings^4^. The transition to legal adulthood and increased autonomy in relation to health care may intensify feelings of dissatisfaction and a critical perception of one's own oral health, especially in the face of access difficulties, previous negative experiences, or intensified aesthetic demands in youth.

Regarding predisposing/facilitating variables (Block 2), socioeconomic status remained associated with the outcome. Precarious housing, analyzed in 2003, and receipt of government benefits in 2023 were markers of social vulnerability with a direct impact on dissatisfaction with oral health. Previous studies have already demonstrated the influence of social inequalities on the perception of health in adults^8^. In adolescents, this relationship seems even more sensitive, given the symbolic weight of living conditions at this stage of development^2^. In fact, social conditions qualify the way individuals feel, think, and act in relation to their own health^5,23^. Furthermore, HRQoL is profoundly impacted by socioeconomic context^6^, which requires the incorporation of intersectoral strategies into public policies. Therefore, it is suggested that oral health care in adolescence go beyond the clinical approach and consider social determinants, recognizing the impact of material and subjective conditions on the construction of the health experience.

Clinical variables (Block 3), i.e., the presence of dental calculus (2003 and 2023), DMFT index (2003), and tooth loss due to caries (2023) were associated with the outcome in the final models. Previous studies have observed that tooth loss is associated with dissatisfaction with oral health^1,4,23^. In fact, tooth loss can impact important actions such as pronunciation, digestion, and chewing, leading to a worsening quality of life^24^. The presence of dental calculus, in turn, is associated with poor oral hygiene, a reality associated with a worse HRQoL^6^ and which compromises self-image, contributing to a negative perception of oral health, as verified in adolescents in Amazonas^14^. Regarding the DMF index, it was observed that the gross effect of this variable disappeared after adjustment in 2023. This result is consistent with the decline observed in this index between 2003 and 2023, pointing to remarkable progress in the oral health of adolescents in the Southeast^3^. Thus, this study suggests that the DMFT, which is a normative clinical parameter, has become a less robust predictor of subjectivity, particularly of the perception of oral health in adolescents in southeastern Brazil, even though it is an important clinical variable.

Proximal variables (Block 4) — self-reported need for treatment, reported dental pain, and, in 2023, time elapsed since the last visit — were associated with the outcome. Studies in adults have observed that positive self-assessment of oral health is the most important predictor of the perception of no need for treatment^5,8^. The results of this study demonstrate that the same is true for adolescents. Furthermore, authors suggest that frequent dental appointments have important psychosocial significance for adolescents^25^. The present study found that, in 2023, intervals longer than one year and shorter than three years since the last appointment were associated with a higher prevalence of the outcome when compared to annual visits to the dentist. This finding is similar to the results found by other authors when analyzing Brazilian adults, but, in this case, longer intervals (≥3 years) between visits to the dentist were associated with poor oral health assessment^5,8^. Thus, for adolescents in the Southeast region of the country, this article suggests that it is important to encourage annual dental check-ups.

The association between pain and negative self-assessment is well known^5,7,8^, but a recent study has shown that, in adolescents, a single episode of dental pain is enough to cause negative impacts throughout adolescence^26^. In addition, pain in adolescents impacts the quality of life of the family^6^, a reality in line with the contemporary concept that the health of children and adolescents affects both the individual and the family^16^.

The study has some limitations. Because it is observational, it does not allow causal relationships to be established. The use of questionnaires may introduce memory and response biases. In addition, the question regarding the outcome differs slightly between surveys, which may have interfered with the responses, and the age group considered (15 to 19 years) does not cover the entire adolescence. In addition, the number of adolescents excluded, 2.9% (86/2,981 in 2003) and 2.3% (21/919 in 2023), may also have impacted the validity of the findings.

A strong point of the study is the careful methodology of SBBrasil, which trains professionals throughout the country to ensure the reliability of the survey. Using this data is a commitment to those involved, aiming to improve future surveys and understanding of collective oral health problems.

The objective of this study was to identify the prevalence of negative self-assessment of oral health among adolescents living in the Southeast region of Brazil and the factors associated with it. The results reveal that a significant portion of respondents assessed their oral health negatively. In 2003, this perception was associated with older age in the age group analyzed, unfavorable socioeconomic status, dental pain, number of missing/filled/decayed teeth, presence of dental calculus, and self-reported need for treatment. In 2023, however, the total number of lost/filled/decayed teeth lost significance, while tooth loss became significant, as did the longer interval since the last dental visit. The null hypothesis that these factors do not impact adolescents’ self-assessment of oral health was therefore rejected. A subtle change in the factors associated with the outcome over these 20 years can be observed, although a multidimensional structure of variables remains.

These results reinforce the relevance of self-assessment as a subjective indicator sensitive to social inequalities and the clinical and psychosocial conditions of adolescents. It is important to emphasize the need for public policies and oral health care strategies to consider these determinants, incorporating intersectoral approaches and actions that prioritize comprehensive care. In addition, the present study suggests that curative dental care should be articulated, in a complementary manner, with health promotion and disease prevention actions aimed at adolescents, considering the values and perceptions of these individuals. It is necessary to consider that the foundation for the prevention of oral diseases is established in adolescence, particularly due to the cumulative nature of caries and periodontal disease.

This analysis demonstrated an association between self-rated health, social variables, and objective and subjective health indicators in adolescents living in southeastern Brazil, as previously reported in other groups^1,4,5,7,8,13,14,23^. Self-assessment is a simple variable to obtain and can be used as a mediator between symptoms (subjective and reported by the individual), clinical signs of oral diseases (normative and determined by clinical evaluation), and consequences on quality of life^2^. In fact, investigating people's perceptions of their oral health has the advantage of applying a single self-assessment question, constituting a potential complementary indicator to clinical evaluation for the planning of dental services and the analysis of public policies.

In addition, adolescents’ perceptions of their oral health can be used for screening purposes, especially in resource-poor settings. Furthermore, investigating the factors that influence self-assessment of oral health contributes to a more comprehensive understanding of this indicator, providing support for the identification of priority groups for health care and the appropriate allocation of public resources.
