# Supplementary material for: Prevalence of negative self-assessment of oral health and associated factors among adolescents from southeastern Brazil in the 2003 and 2023 Brazil Oral Health surveys
Source: Rev Bras Epidemiol. 2026 Apr 17;29(Suppl 1):e260001supl1. doi: 10.1590/1980-549720260001.supl.1 (PMC13089317; doi:10.1590/1980-549720260001.supl.1)
Supplement: Translation

# Translation: Prevalência da autoavaliação negativa da saúde bucal e fatores associados em adolescentes do Sudeste brasileiro nos levantamentos Saúde Bucal Brasil 2003 e 2023

**DOI:** 10.1590/1980-549720250001.supl.1.2

**Authors:** Sheyla Omonte Neves, Giana Zarbato Longo, Karla Maria Damiano Teixeira

**Affiliations:** I Universidade Federal de Viçosa – Viçosa (MG), Brasil.; II Universidade Federal do Rio Grande do Norte – Natal (RN), Brasil.

**Keywords:** Qualidade de vida, Saúde pública, Odontologia, Inquéritos epidemiológicos, Fatores de risco

## Abstract

**Objetivo:**

Determinar a prevalência e os fatores associados à autoavaliação negativa da saúde bucal (ANSB) entre adolescentes residentes na região Sudeste do Brasil.

**Métodos:**

Trata-se de um estudo transversal com dados da Pesquisa Nacional de Saúde Bucal de 2003 e 2023. Por meio de exame e questionário, foram obtidos dados clínicos, socioeconômicos e de percepções de saúde bucal de 2.981 jovens em 2003, e 919 em 2023. Foram realizadas análises descritiva, bivariada e multivariada, por meio de regressão de Poisson com variância robusta.

**Resultados:**

A ANSB foi observada em 43,9% (IC95% 38,7–49,1) dos participantes em 2003 e em 33,4% (IC95% 27,2–40,3) em 2023. Na análise multivariada, os fatores associados a pior autoavaliação da saúde bucal foram: ter 18 ou 19 anos (RP 1,04; IC95% 1,01–1,08 em 2003 e RP 1,27; IC95% 1–1,63 em 2023); não possuir casa própria (RP 1,16; IC95% 1,01–1,32 em 2003); receber auxílio governamental (RP 1,40; IC95% 1,11–1,77); sentir necessidade de tratamento (RP 2,7; IC95% 1,94–3,74 em 2003 e RP 3; IC95% 1,58–5,70 em 2023); sentir dor dentária (RP 1,67; IC95% 1,38–2,04 em 2003 e RP 1,72; IC95% 1,17–2,54 em 2023); apresentar cálculo dentário (RP 1,22; IC95% 1,04–1,43 em 2003 e RP 1,84; IC95% 1,28–2,66 em 2023); ter dentes perdidos, cariados ou obturados (RP 1,02; IC95% 1,01–1,04 em 2003); ter extraído dentes devido à cárie (RP 2,19; IC95% 1,42–3,37 em 2023); e ter visitado o dentista há 2 a 3 anos (RP 1,89; IC95% 1,39–2,56 em 2023).

**Conclusão:**

Observou-se uma elevada prevalência da ANSB entre adolescentes do Sudeste, associada a uma estrutura multidimensional de variáveis.

## INTRODUÇÃO

A autoavaliação da saúde é uma variável subjetiva que se relaciona com condições clínicas e indicadores de morbidade e mortalidade^1^. É uma maneira simples de compreender a multidimensionalidade relacionada à perspectiva do indivíduo sobre a própria saúde^2^. No âmbito da saúde bucal, a autoavaliação reflete a Qualidade de Vida Relacionada à Saúde Bucal (QVRSB), associando-se à condição objetiva da saúde e à funcionalidade, além de incorporar valores culturais, políticos, econômicos e sociais^1,3–8^. Assim, observa-se a saúde bucal de maneira mais ampla, reconhecendo a sua complexidade e o impacto das desigualdades em saúde bucal e dos determinantes sociais associados^6^. A literatura mostra que não brancos^9^, com renda domiciliar per capita de até um salário mínimo^10^ e classes sociais menos privilegiadas^10,11^ apresentam maior proporção de avaliação negativa da saúde na população brasileira. Estudos também destacam que condições socioeconômicas são importantes determinantes de saúde das populações^10,11^.

Em 2004, a implantação do programa Brasil Sorridente ampliou o acesso dos brasileiros aos serviços de saúde bucal^12^. A análise do ciclo dessa política aponta avanços nos indicadores de saúde, expansão da rede de serviços odontológicos e aumento da cobertura assistencial^12^. Por outro lado, o comprometimento da qualidade de vida, refletido na autoavaliação da saúde bucal, manteve-se elevado em estudos realizados após a implementação do Brasil Sorridente^4,5,13,14^.

Nesse contexto, a autoavaliação da saúde bucal pode constituir um significativo complemento aos índices clínicos, pois considera os elementos biológicos, socioeconômicos e culturais que impactam o cuidado em saúde bucal e o processo saúde-doença^1,10,14^. Essa variável assume especial relevância na adolescência, fase da vida em que geralmente se inicia a responsabilidade pelo autocuidado e se consolidam hábitos alimentares e de higiene^15^. Além disso, por se tratar de uma fase de transição marcada pelo entrelaçamento entre dependência e independência, a adolescência favorece o surgimento de comportamentos potencialmente prejudiciais à saúde^6^. Ademais, condições que afetam negativamente o bem-estar do jovem podem repercutir na qualidade de vida de suas famílias^16^.

Cabe ressaltar que a heterogeneidade regional, com suas diferenças culturais e socioeconômicas, compromete a generalização de resultados de abrangência nacional para indivíduos de uma região do país^10^. Isso reforça a importância de informações em saúde de maneira regionalizada. Assim, o objetivo deste estudo foi avaliar a prevalência da autoavaliação negativa da saúde bucal (ANSB) entre jovens brasileiros de 15 a 19 anos, residentes na região Sudeste, e investigar sua associação com condições clínicas, demográficas, de predisposição/facilitação (como renda e cuidado em saúde), de percepção e necessidade de tratamento. A hipótese nula é de que esses fatores não influenciam a autoavaliação da saúde bucal dos jovens. Há estudos realizados em outras regiões do país e em outras faixas etárias sobre o tema^4,5,13,14^, mas este é o primeiro que analisa o jovem do Sudeste. Ademais, a literatura aponta que esse tema ainda é pouco explorado em pesquisas nacionais e internacionais^4^, o que reforça a importância desta investigação.

## MÉTODOS

Trata-se de um estudo transversal que incluiu indivíduos de 15 a 19 anos, residentes no Sudeste do Brasil, participantes do levantamento epidemiológico Saúde Bucal Brasil 2003 (SBBrasil 2003) ou 2023 (SBBrasil 2023). Foram excluídos aqueles cujos dados em relação à autoavaliação de saúde bucal estavam incompletos. O Projeto SBBrasil possui abrangência nacional e representatividade para as capitais estaduais, o Distrito Federal e as cinco regiões geográficas (Norte, Nordeste, Sudeste, Sul e Centro-Oeste). O planejamento considerou a seleção de uma amostra probabilística por conglomerados. Foram realizados treinamento e calibração das equipes odontológicas. Detalhes sobre desenho, cálculo amostral, calibração e coleta dos dados podem ser encontrados nos relatórios oficiais^17,18^. A coleta ocorreu entre os anos de 2002 e 2003^17^ e de 2022 a 2024^18^. O SBBrasil 2003 foi aprovado pela Comissão Nacional de Ética em Pesquisa (Conep), conforme parecer n^o^ 581/2000^17^. Já o SBBrasil 2023 foi aprovado conforme parecer Conep n^o^ 4.823.054/2021^18^.

Os agravos bucais examinados incluíram: prevalência de cárie pelo índice de Dentes Cariados, Perdidos e Obturados (CPO-D); condição periodontal pelo índice Periodontal Comunitário (CPI), mensurando sangramento, cálculo e presença de bolsas periodontais; e oclusão dentária via índice de Estética Dental (DAI), que combina estado oclusal e necessidade ortodôntica^17,18^. Foram também coletados, por meio de questionário aplicado durante a visita domiciliar, dados socioeconômicos, demográficos, de morbidade bucal referida, de acesso/uso a serviços de saúde bucal e de autopercepção em saúde bucal^17,18^.

A variável dependente foi a autoavaliação da saúde bucal. Em 2003, foi avaliada pela pergunta: "*Como classificaria a sua saúde bucal?*", com cinco opções de resposta: "péssima", "ruim", "regular", "boa" e "ótima". Respostas como "sem informação" ou "não sabe/não informou" foram excluídas da análise. Em 2023, perguntou-se: "*Em geral, como avalia a sua saúde bucal (dentes e gengivas)?*", com respostas: "muito boa", "boa", "regular", "ruim", "muito ruim" e "não sabe/não respondeu", sendo esta última excluída da análise. Para fins analíticos, as respostas foram agrupadas em duas categorias: positiva (ótima, muito boa ou boa) e negativa (regular, ruim, muito ruim ou péssima), conforme utilizado em estudos anteriores^1,4,8,13^. Há diferença acentuada na pergunta e nas opções de resposta sobre autoavaliação da saúde bucal no questionário do SBBrasil 2010, em relação aos demais SBBrasil. Assim, optou-se por não analisar os dados do SBBrasil 2010 neste trabalho.

As variáveis independentes deste trabalho foram selecionadas com base no modelo hierárquico de Gift et al.^19^, com pequenas adaptações, assim como fizeram outros autores^5^. O modelo conceitual teoriza que a autoavaliação da saúde bucal é uma função múltipla, incluindo características demográficas, fatores de predisposição/facilitação, fatores da condição de saúde bucal e fatores de autopercepção da necessidade de tratamento^19^.

Similarmente, as variáveis independentes deste trabalho foram organizadas em 4 blocos (Figura 1):

**Figura 1:** Modelo teórico de determinação das variáveis independentes adaptado do modelo proposto por Gift et al.^19^

Blocos 1 e 2: variáveis demográficas e de predisposição/facilitação;

Bloco 3: condições clínicas de saúde bucal; e

Bloco 4: variáveis de necessidade de tratamento e percepção.

Dos mais de 30 indicadores disponíveis nos bancos de dados dos SBBrasil 2003 e SBBrasil 2023, foram selecionadas as variáveis mais representativas da saúde bucal e da QVRSB, considerando a literatura atual e o modelo conceitual adotado, que contempla os determinantes sociais e biológicos da saúde^5,19^.

A análise estatística foi realizada utilizando o software *Stata* ^®^, versão 17.0, com o módulo *survey*, seguindo as diretrizes recomendadas para amostras complexas^20^. Inicialmente, realizou-se a descrição da amostra, com apresentação de proporções, médias e Intervalos de Confiança de 95% (IC95%). Em seguida, foram conduzidas análises bivariadas da prevalência do desfecho, sendo considerada autoavaliação negativa como categoria de referência da variável dependente, calculando-se as Razões de Prevalência (RP) e IC95%. Para testar o efeito das variáveis independentes no desfecho foi utilizado o modelo de regressão de Poisson com variância robusta, indicada quando a prevalência do desfecho ultrapassa 20%^21^. Na análise simples verificou-se a associação entre cada variável independente e o desfecho, calculando-se RP e IC95%. Variáveis com valor de p<0,25 nas análises brutas foram incluídas no modelo múltiplo^22^. Ao final da análise múltipla, as variáveis com p<0,05 permaneceram no modelo final de cada bloco e foram consideradas fatores de ajuste para os blocos subsequentes, não sendo acompanhados de teste de *deviance* ou Pearson para avaliação de ajuste.

### Declaração de disponibilidade de dados

Dados disponíveis mediante solicitação: Todo o conjunto de dados que dá suporte aos resultados deste estudo está disponível mediante solicitação ao Ministério da Saúde. Para ter acesso aos bancos de dados do SB Brasil, é necessário concordar com o termo de compromisso e preencher os dados solicitados pelo Ministério da Saúde.

## RESULTADOS

O Projeto SBBrasil coletou dados de 16.832 e 8.054 indivíduos de 15 a 19 anos no SBBrasil 2003 e 2023, respectivamente. Na região Sudeste, a amostra foi composta por 2.981 adolescentes em 2003 e 919 em 2023. Destes, 86 indivíduos foram excluídos em 2003 (informações incompletas quanto à autoavaliação da saúde bucal), resultando em uma amostra de 2.895 adolescentes (97,1%). Em 2023, 21 indivíduos foram excluídos pelo mesmo motivo, totalizando 898 indivíduos incluídos na análise (97,7%).

A Tabela 1 apresenta a prevalência da ANSB, que foi de 43,9% (IC95% 38,7–49,1) nos participantes do SBBrasil 2003 e de 33,4% (IC95% 27,2–40,3) no SBBrasil 2023. No ano mais recente, 35,1% (IC95% 27,9–43) dos adolescentes pertenciam a famílias beneficiárias de programas de transferência de renda, como o Bolsa Família ou o Benefício de Prestação Continuada (BPC), proporção semelhante à observada entre os que não possuíam moradia própria em 2003 (20,3%; IC95% 14,5–28,1).

**Tabela 1:** Distribuição e prevalência da Autoavaliação Negativa da Saúde Bucal (ANSB) segundo características da amostra dos adolescentes (15 a 19 anos) nos períodos de 2003 e 2023. Projeto SBBrasil, MS, Brasil.

		Distribuição da amostra (n=2.895)		Prevalência ANSB		Distribuição da amostra (n=898)		Prevalência ANSB	
		2003				2023			
		%-média	IC95%	%-média	IC 95%	%-média	IC95%	%-média	IC95%
Total				43,9	38,7–49,1			33,4	27,2–40,3
Sexo									
	Masculino	42,2	37,6–46,9	43,1	37,1–49,3	51,3	45,3–57,2	33,2	22,2–46,3
	Feminino	57,8	53,1–62,3	44,3	39,2–49,6	48,6	42,7–54,6	33,7	26,8–41,3
Idade (anos)									
	15–17	64,3	59,9–68.4	39,2	33,8–44,8	65,7	57,3–73,1	29,8	20,7–41
	18–19	35,7	31,6–40	52,2	46,3–58,1	34,3	26,9–42,6	40,3	33,4–47,8
Cor da pele									
	Branca	41,1	31,9–50,8	40,2	33,4–47,2	46	36–56,1	27,5	19,4–37,5
	Não branca	58,9	49,2–68,1	46,4	40,9–52	54	43,8–63,9	38,2	31,7–45,1
Recebe benefício									
	Não	-	-	-	-	64,8	56,9–72	26,6	20,8–33,2
	Sim					35,1	27,9–43	46,9	37,4–56,7
Moradia própria									
	Sim	79,7	72,4–85,5	43,4	38,5–48,3	-	-	-	-
	Não	20,3	14,5–28,1	45,8	36,6–55,2				
Plano odontológico									
	Sim	9,7	7,5–12,4	42,9	30,1–56,7	16,9	9,9–27,3	18,8	11,8–28,8
	Não	90,3	87,6–92,4	43,9	38,8–49,3	83,1	72,7–90,1	36,7	29,2–44,9
Dentes perdidos									
	Nenhum	80,2	77–83	39,6	33,2–46,4	92,3	88,8–94,7	30,3	24,4–36,9
	1 ou mais	19,8	16,9–23	61	55,2–66,5	7,7	5,2–11,2	71,2	54,5–83,6
Cálculo dental									
	Sextantes hígidos	65,8	54,2–75,8	36,5	29,9–43,6	67,8	59,5–75,1	24,6	19,7–30,3
	Presença de cálculo	34,2	24,2–45,8	54,1	47,9–60,3	32,1	24,8–40,5	51,5	38,9–63,9
Sangramento gengival									
	Sextantes hígidos	74,6	63,6–84,2	36,5	30–43,6	68,9	61,4–75,6	27,3	20,6–35,1
	Presença de sangramento	25,4	16,7–36,4	49,3	41,5–57,1	31	24,4–38,6	46,9	38,4–55,7
	CPOD (média)	4,9	4,5–5,4	5,8	5,3–6,3	2,8	2,2–3,5	4,1	2,8–5,5
DAI									
	Sem oclusopatia	43,5	38,4–48,8	44,9	38–51,9	72,5	65,2–78,1	30,1	23,2–38,1
	Oclusopatia definida	22,5	20,1–25	39,9	34,8–45,1	14	9,8–19,7	36,3	21,8–53,7
	Oclusopatia grave	14,9	12,3–17,8	44,8	38,7–51,1	6,1	4,4–8,3	34,2	22,1–48,8
	Muito grave	19,1	16,9–21,5	45,2	35,3–55,6	7,3	4,5–11,6	58,5	41,6–73,6
Necessidade de tratamento									
	Não	26,3	20,7–32,7	17,8	12,9–23,8	40,8	32,3–49,8	11,7	6,6–19,9
	Sim	73,7	67,2–79,3	53,2	47,7–58,5	59,2	50,1–67,7	48,9	41,5–56,3
Tempo (anos)*									
	Até 1	44,6	39,5–49,8	35,3	29–42,1	53,4	45,6–60,9	25,9	18,3–35,2
	2 a 3	24	19,3–29,4	47,5	41,8–53,3	35,1	27,4–43,7	42,5	34,4–51
	Mais de 3 ou nunca	31,4	27,2–36,2	53,3	46,0–60,4	11,5	7,8–16,7	34,6	23,5–47,7
Tipo do serviço†									
	Particular	38,4	34,8–42,1	41,6	34,7–48,8	61,9	56,2–67,4	27,9	20,2–37,2
	Público	61,6	57,9–65,2	44,2	39–48,5	38	32,6–43,8	40,5	29,5–53
Dor (últimos 6 meses)									
	Não	64,3	58,6–69,5	32,3	26,5–38,7	80,9	75,8–85,1	27,6	22,1–33,7
	Sim	35,7	30,5–41,4	64,7	59,2–69,8	19,1	14,8–24,2	58,7	37,3–77,2

ANSB: Autoavaliação Negativa da Saúde Bucal.

* tempo decorrido desde a última consulta odontológica;

† o tipo de serviço utilizado na última consulta odontológica.

A Tabela 2 exibe as razões de prevalência brutas e ajustadas para cada variável independente, bem como os modelos obtidos na análise múltipla, estratificada por ano do estudo. Em 2003, após o ajuste entre as variáveis do Bloco 1, os adolescentes com idades entre 18 e 19 anos apresentaram maior prevalência de autoavaliação negativa da saúde bucal. No Bloco 2, após ajuste mútuo e controle pelas variáveis do Bloco 1, a ausência de moradia própria manteve-se associada ao desfecho. Entre as variáveis clínicas (Bloco 3), ter dentes perdidos, cariados e obturados e a presença de cálculo dentário mantiveram-se significativamente associadas à percepção negativa da saúde bucal. No Bloco 4, reportar dor de dente nos últimos seis meses e a necessidade autorreferida de tratamento apresentaram associação estatisticamente significativa mesmo após os ajustes.

**Tabela 2:** Análises univariadas e ajustadas, Razões de Prevalência e Intervalos de Confiança de 95% obtidos por análise de regressão de Poisson para associação entre a autoavaliação negativa da saúde bucal e as variáveis independentes, da amostra dos adolescentes (15 a 19 anos) nos períodos de 2003 e 2023. Projeto SBBrasil, MS, Brasil.

Variáveis		2003		2023	
		RP bruta (IC95%)	RP ajustada (IC95%)	RP bruta (IC95%)	RP ajustada (IC95%)
Sexo					
	Masculino	1		1	
	Feminino	1,02 (0,94–1,13)		1,01 (0,64–1,61)	
Idade (anos)					
	15–17	1	1	1	1
	18–19	1,10 (1,07–1,1,4)	1,04 (1,01–1,08)	1,35 (0,88–2,07)	1,27 (1,00–1,62)
Cor de pele					
	Branca	1		1	
	Não branca	1,16 (0,98–1,36)		1,38 (0,97–1,97)	
Recebe benefício					
	Não			1	1
	Sim			1,77 (1,37–2,27)	1,40 (1,11–1,77)
Moradia própria					
	Sim	1	1		
	Não	1,06 (0,93–1,25)	1,16 (1,01–1,32)		
Plano odontológico					
	Sim	1		1	
	Não	1,03 (0,75–1,40)		1,95 (1,08–3,49)	
Dentes perdidos					
	Nenhum	1		1	1
	1 ou mais	1,54 (1,26–1,88)		2,35 (1,77–3,11)	2,19 (1,42–3,37)
Cálculo dental					
	Sextantes hígidos	1	1	1	1
	Presença de cálculo	1,48 (1,22–1,80)	1,22 (1,04–1,43)	2,09 (1,51–2,90)	1,84 (1,28–2,66)
Sangramento gengival					
	Sextantes hígidos	1		1	
	Presença de sangramento	1,35 (1,08–1,68)		1,72 (1,27–2,32)	
CPOD (média)		1 1,04 (1,02–1,06)	1 1,02 (1,01–1,04)	1 1,06 (1,03–1,09)	
DAI					
	Sem oclusopatia	1		1	
	Oclusopatia definida	0,89 (0,76–1,04)		1,20 (0,74–1,95)	
	Oclusopatia grave	0,99 (0,83–1,20)		1,13 (0,72–1,81)	
	Muito grave	1,01 (0,78–1,29)		1,94 (1,33-2,84)	
Necessidade de tratamento					
	Não	1	1	1	1
	Sim	2,99 (2,22–4,03)	2,70 (1,94–3,74)	4,17 (2,31–7,53)	3,00 (1,58–5,70)
Tempo (anos)*					
	Até 1	1		1	1
	2 a 3	1,34 (1,12–1,61)		1,64 (1,21–2,23)	1,89 (1,39–2,56)
	> 3 ou nunca	1,51 (1,26–1,80)		1,34 (0,86–2,08)	1,16 (0,76–1,77)
Tipo do serviço†					
	Particular	1		1	
	Público	1,06 (0,92–1,23)		1,45 (0,97–2,15)	
Dor de dente					
	Não	1	1	1	1
	Sim	2,00 (1,63–2,47)	1,67 (1,38–2,04)	2,13 (1,48–3,05)	1,72 (1,17–2,54)

RP: Razões de Prevalência; IC95%: Intervalos de Confiança de 95%.

* tempo decorrido desde a última consulta odontológica;

† o tipo de serviço utilizado na última consulta odontológica.

Em 2023, as variáveis idade (18 ou 19 anos), recebimento de auxílio governamental, perda dentária por cárie, presença de cálculo dentário, necessidade percebida de tratamento odontológico, tempo desde a última consulta odontológica (de 2 a 3 anos), e dor de dente recente mostraram-se positivamente associadas à insatisfação com a saúde bucal, com significância estatística, conforme demonstrado na Tabela 2.

## DISCUSSÃO

No que tange aos jovens de 15 a 19 anos residentes na região Sudeste do Brasil, os dados apresentados evidenciam queda em importantes indicadores de 2003 para 2023, como no índice CPO-D e na dor dentária, alinhando-se com a bem-sucedida implementação do Brasil Sorridente, que proporcionou melhorias estruturais e organizacionais no sistema de saúde bucal nos seus 20 anos de existência^12^. Entretanto, a prevalência da ANSB manteve-se acima de 30%. Não obstante, houve uma queda nesta variável (de 43,9% em 2003 para 33,4% em 2023), mas sem significância estatística. Estudos conduzidos em adolescentes de outras regiões, como nas cidades de Santa Maria (RS)^4^ e Manaus (AM)^14^, também observaram elevada insatisfação com a saúde bucal, sugerindo que, apesar dos avanços estruturais e assistenciais, os indicadores subjetivos entre adolescentes permanecem críticos.

Contudo, estudos com adultos e idosos apresentam, em geral, autoavaliações mais positivas^1,5,13,23^, o que pode refletir diferenças metodológicas ou, mais provavelmente, distintas construções perceptivas da saúde ao longo do ciclo de vida. Fatores emocionais, culturais e sociais modulam essas percepções, sendo mais agudamente sentidos durante a adolescência, período de intensas transformações psicossociais^15^. Assim, argumenta-se que os avanços alcançados pelo Brasil Sorridente precisam ser acompanhados de estratégias que incorporem os determinantes subjetivos da saúde bucal, especialmente na juventude.

Dentre as variáveis demográficas (Bloco 1), a idade manteve associação significativa com o desfecho no modelo ajustado. A prevalência de ANSB aumentou entre os adolescentes mais velhos (18–19 anos), replicando achados anteriores^4^. A transição para a maioridade legal e o aumento da autonomia em relação ao cuidado com a saúde podem intensificar sentimentos de insatisfação e percepção crítica do próprio estado bucal, em especial diante de dificuldades de acesso, experiências negativas prévias ou demandas estéticas intensificadas na juventude.

No que tange às variáveis de predisposição/facilitação (Bloco 2), a situação socioeconômica manteve-se associada ao desfecho. A precariedade habitacional, analisada em 2003, e o recebimento de benefícios governamentais, em 2023, configuraram-se como marcadores de vulnerabilidade social com impacto direto na insatisfação com a saúde bucal. Estudos prévios já demonstraram a influência das desigualdades sociais na percepção da saúde em adultos^8^. Em adolescentes, essa relação parece ainda mais sensível, dado o peso simbólico das condições de vida nesse estágio de desenvolvimento^2^. De fato, as condições sociais qualificam a maneira como os indivíduos sentem, pensam e agem em relação à própria saúde^5,23^. Ademais, a QVRSB é profundamente impactada pelo contexto socioeconômico^6^, o que exige a incorporação de estratégias intersetoriais às políticas públicas. Portanto, sugere-se que o cuidado em saúde bucal na adolescência vá além da abordagem clínica e contemple os determinantes sociais, reconhecendo o impacto das condições materiais e subjetivas na construção da experiência de saúde.

As variáveis clínicas (Bloco 3), ou seja, presença de cálculo dentário (2003 e 2023), índice CPO-D (2003) e perda dentária por cárie (2023), apresentaram associação com o desfecho nos modelos finais. Estudos anteriores observaram que ter perdido dentes está associado à insatisfação com a saúde bucal^1,4,23^. De fato, a perda dentária pode impactar ações importantes como pronúncia, digestão e mastigação, relacionando-se a uma piora de qualidade de vida^24^. A presença de cálculo dentário, por sua vez, está associada à higiene oral deficiente, realidade associada a uma pior QVRSB^6^ e que compromete a autoimagem, contribuindo para a percepção negativa da saúde bucal, conforme verificado em adolescentes do Amazonas^14^. Em relação ao índice CPO-D, observou-se que o efeito bruto desta variável desapareceu após o ajuste em 2023. Esse resultado é compatível com a queda observada neste índice entre 2003 e 2023, apontando notável avanço na saúde bucal de adolescentes no Sudeste^3^. Assim, este estudo sugere que o CPO-D, que é um parâmetro clínico normativo, tornou-se um preditor pouco robusto da subjetividade, em particular da percepção de saúde bucal em adolescentes do Sudeste brasileiro, ainda que seja uma importante variável clínica.

As variáveis proximais (Bloco 4) – necessidade autorreferida de tratamento, dor dentária reportada e, em 2023, tempo decorrido desde a última consulta – apresentaram associação com o desfecho. Estudos no adulto observaram que a autoavaliação da saúde bucal positiva é o mais importante preditor da percepção de não necessidade de tratamento^5,8^. Os resultados deste estudo demonstram que o simétrico é verdadeiro para a adolescência. Ademais, autores sugerem que consultas odontológicas frequentes apresentam um significado psicossocial importante para o adolescente^25^. O presente estudo verificou que, em 2023, intervalos maiores do que 1 ano e menores do que 3 anos da última consulta estavam associados a maior prevalência do desfecho, quando comparados à visita anual ao dentista. Tal achado se assemelha aos resultados encontrados por outros autores ao analisarem adultos brasileiros, mas nesse caso observaram-se intervalos mais longos (≥3 anos) de visita ao dentista associando-se à avaliação ruim da saúde bucal^5,8^. Assim, para os adolescentes da região Sudeste do país, este artigo sugere ser importante incentivar a periodicidade anual do acompanhamento odontológico.

É conhecida a associação entre dor e autoavaliação negativa^5,7,8^, mas um estudo recente demonstrou que, no adolescente, basta um episódio de dor dentária para que impactos negativos acompanhem toda a adolescência^26^. Além disso, a dor no adolescente impacta também a qualidade de vida da família^6^, realidade alinhada ao conceito contemporâneo de que a saúde da criança e do adolescente afeta tanto o indivíduo quanto a família^16^.

O estudo apresenta algumas limitações. Por ser observacional, não permite estabelecer relações de causalidade. O uso de questionários pode introduzir vieses de memória e resposta. Ademais, a pergunta referente ao desfecho difere ligeiramente entre os levantamentos, o que pode ter interferido nas respostas, e a faixa etária considerada (15 a 19 anos) não abarca toda a adolescência. Além disso, o número de adolescentes excluídos, 2,9% (86/2.981 em 2003) e 2,3% (21/919 em 2023), pode também ter impactado a validade dos achados.

Como ponto forte do estudo destaca-se a metodologia cuidadosa do SBBrasil, capacitando profissionais em todo o país em prol da confiabilidade do levantamento. Utilizar esses dados é um compromisso com os envolvidos, visando à melhoria dos levantamentos futuros e compreensão dos problemas de saúde bucal coletiva.

O objetivo deste estudo foi identificar a prevalência da autoavaliação negativa da saúde bucal entre adolescentes residentes na região Sudeste do Brasil e os fatores a ela associados. Os resultados revelam que uma parcela expressiva avaliou negativamente sua saúde bucal. Em 2003, essa percepção esteve associada à idade mais avançada na faixa etária analisada, à condição socioeconômica desfavorável, à dor dentária, ao número de dentes perdidos/obturados/cariados, à presença de cálculo dentário e à necessidade autorreferida de tratamento. Em 2023, por sua vez, o número total de dentes perdidos/obturados/cariados perdeu significância, enquanto a perda dentária se tornou significativa, assim como o maior intervalo desde a última consulta odontológica. A hipótese nula de que esses fatores não impactam a autoavaliação de saúde bucal do adolescente foi, portanto, rejeitada. Observa-se uma mudança sutil nos fatores associados ao desfecho ao longo desses 20 anos, embora permaneça uma estrutura multidimensional de variáveis.

Tais resultados reforçam a relevância da autoavaliação como indicador subjetivo sensível às desigualdades sociais e às condições clínicas e psicossociais dos adolescentes. Ressalta-se a necessidade de que políticas públicas e estratégias de cuidado em saúde bucal considerem esses determinantes, incorporando abordagens intersetoriais e ações que priorizem a integralidade do cuidado. Além disso, o presente estudo sugere que a assistência odontológica curativa seja articulada, de maneira complementar, a ações de promoção de saúde e prevenção da doença voltadas para o adolescente, considerando os valores e percepções desses indivíduos. É preciso ponderar que o alicerce da prevenção de doenças bucais se estabelece na adolescência, em particular pelo caráter cumulativo da cárie e da doença periodontal.

Esta análise demonstrou associação entre a autoavaliação em saúde, variáveis sociais e indicadores objetivos e subjetivos de saúde no adolescente residente no Sudeste brasileiro, conforme já reportado anteriormente em outros grupos^1,4,5,7,8,13,14,23^. A autoavaliação é uma variável simples de obter e pode ser utilizada como mediadora entre os sintomas (subjetivos e referidos pelo indivíduo), os sinais clínicos das doenças bucais (normativos e determinados pela avaliação clínica) e as consequências na qualidade de vida^2^. De fato, investigar a percepção das pessoas sobre sua saúde bucal apresenta a vantagem de se aplicar uma única questão de autoavaliação, constituindo um potencial indicador complementar à avaliação clínica para o planejamento dos serviços odontológicos e a análise de políticas públicas.

Além disso, as percepções dos adolescentes sobre sua saúde bucal podem ser utilizadas para fins de triagem, especialmente em contextos com escassez de recursos. Ademais, investigar os fatores que influenciam a autoavaliação da saúde bucal contribui para a compreensão mais abrangente desse indicador, oferecendo subsídios para a identificação de grupos prioritários para a atenção à saúde e para o adequado direcionamento de recursos públicos.
